# When agriculture meets biotechnology: a route for future agricultural innovation

**DOI:** 10.1007/s44307-024-00047-3

**Published:** 2024-11-01

**Authors:** Jun-Peng Shi, Ruo-Han Xie, Fang Yang, Wang Zhou, Xiao-Qian Jiang, Hai-Fei Hu, Chao Yang, Yong-Yao Xie, Shi Xiao

**Affiliations:** 1 State Key Laboratory of Biocontrol, Guangdong Provincial Key Laboratory of Plant Stress Biology, School of Agriculture and Biotechnology, Sun Yat-Sen University, Shenzhen, 518107 China; 2 Rice Research Institute, Guangdong Academy of Agricultural Sciences, Guangzhou, 510640 China; 3 Guangdong Provincial Key Laboratory of Applied Botany, South China Botanical Garden, Chinese Academy of Sciences, Guangzhou, 510650 China; 4 College of Agriculture, South China Agricultural University, Guangzhou, 510642 China

The application of biotechnology plays a profound role in advancing the development of agricultural science and technology. In recent decades, remarkable progress has been witnessed in biotechnology, including molecular biology, genetic engineering, male sterility technology, and biopesticides. These advancements have found successful applications across various domains of agricultural researches, encompassing gene cloning for important agronomic traits, accelerated improvement of complex traits through transgenic approaches or harnessing hybrid vigor, as well as effective management of agricultural pests and diseases. Given the exponential growth of the global population and ongoing climate change challenges, we anticipate an increasingly prominent role for biotechnology in supporting agricultural research to sustainably nourish an estimated population exceeding 9 billion individuals by 2050.

The agricultural research at Sun Yat-sen University (SYSU) boasts a rich history, dating back to its establishment in 1924 by Dr. Sun Yat-sen. During the early stages of SYSU's development, Prof. Ying Ding from the School of Agriculture achieved a significant milestone by successfully developed 'Zhongshan No.1', the world's first rice variety incorporating wild rice lineage. Another notable contribution came from Prof. Yaoxiang Huang, who pioneered semi-dwarf rice breeding and introduced an important dwarf variety known as 'Guang Square Dwarf'. His groundbreaking work earned him recognition as the pioneer of semi-dwarf rice by the International Rice Research Institute, subsequently sparking the 'Green Revolution' with numerous identified semi-dwarf rice varieties. Additionally, Prof. Zhelong Pu led pioneering research on 'Biological Control of Pests', earning him acclaim as the father of biological pest control in southern China. In 1952, SYSU's School of Agriculture underwent reorganization into South China Agricultural College (now South China Agricultural University) and Central China Agricultural College (Huazhong Agricultural University). To address food security concerns and promote agricultural modernization, SYSU re-established the School of Agriculture in 2018 which was later renamed as the School of Agriculture and Biotechnology in 2024 to better align with discipline development and regional agricultural industries.

We are currently advancing the application of cutting-edge biotechnologies, such as gene editing, synthetic biology, multi-omics, and artificial intelligence (AI), in various agricultural research fields including Plant and Animal breeding, Plant Protection, Pests Management and Control, Smart Agriculture, Sustained Agriculture, and Agricultural Resource Utilization. For instance, Chinese scientists at CAAS have successfully employed synthetic biology techniques to reconstruct the biosynthetic pathway of paclitaxel precursor in vitro (Jiang et al. 2024). Notable achievements also include utilizing gene editing technology to enhance yield and resistance against powdery mildew in wheat by modifying the *MLO* gene (Li et al. 2022); furthermore, AI has been implemented for protein structure prediction and novel protein design (Abramson et al. 2024; Notin et al. 2024).

To further advance research in agricultural fields by utilizing state-of-the-art biotechnologies and commemorate the 100th anniversary of the agriculture discipline at SYSU, we have launched a special issue of "Agriculture and Biotechnology" to publish cutting-edge research progress in related areas. Currently, this special issue has published six papers (including one Editorial, four Reviews, and one Research Article), showcasing advancements in crop genomics (Zhu et al. 2024), genetic control of plant development (Li and Su 2024), nutrient modulation (Li et al. 2024), transformation (Wu et al. 2024) and other complex traits (Liu et al. 2024), as well as the utilization of arbuscular mycorrhizal fungi (AMF) in paddy fields (Cao et al. 2024). We eagerly anticipate the forthcoming publication of additional papers in our journal to promote the integration of *Advanced Biotechnology* with agricultural research for ensuring food security, fostering sustainable development, and maintaining ecological balance and safety.
